# The Role of UV-B light on Small RNA Activity During Grapevine Berry Development

**DOI:** 10.1534/g3.118.200805

**Published:** 2019-01-15

**Authors:** Sukumaran Sunitha, Rodrigo Loyola, José Antonio Alcalde, Patricio Arce-Johnson, José Tomás Matus, Christopher D. Rock

**Affiliations:** * Department of Biological Sciences, Texas Tech University, Lubbock TX 79409-3131,; † Departamento de Genética Molecular y Microbiología, Pontificia Universidad Católica de Chile, Santiago, Chile; ‡ Departamento de Fruticultura y Enología, Pontificia Universidad Católica de Chile, Santiago, Chile; § Center for Research in Agricultural Genomics (CRAG) CSIC-IRTA-UAB-UB, Barcelona, Spain

**Keywords:** veraison, miRNAs, siRNAs, oxidative stress, anthocyanin, polyphenols, MYBA, light signaling

## Abstract

We explored the effects of ultraviolet B radiation (UV-B) on the developmental dynamics of microRNAs and phased small-interfering-RNA (phasi-RNAs)-producing loci by sequencing small RNAs in vegetative and reproductive organs of grapevine (*Vitis vinifera* L.). In particular, we tested different UV-B conditions in *in vitro*-grown plantlets (high-fluence exposition) and in berries from field-grown (radiation filtering) and greenhouse-grown (low- and high-fluence expositions) adult plants throughout fruit development and ripening. The functional significance of the observed UV-coordinated miRNA responses was supported by degradome evidences of ARGONAUTE (AGO)-programmed slicing of mRNAs. Co-expression patterns of the up-regulated miRNAs miR156, miR482, miR530, and miR828 with cognate target gene expressions in response to high-fluence UV-B was tested by q-RT-PCR. The observed UV-response relationships were also interrogated against two published UV-stress and developmental transcriptome datasets. Together, the dynamics observed between miRNAs and targets suggest that changes in target abundance are mediated transcriptionally and, in some cases, modulated post-transcriptionally by miRNAs. Despite the major changes in target abundance are being controlled primarily by those developmental effects that are similar between treatments, we show evidence for novel miRNA-regulatory networks in grape. A model is proposed where high-fluence UV-B increases miR168 and miR530 that target ARGONAUTE 1 (AGO1) and a *Plus-3 domain* mRNA, respectively, while decreasing miR403 that targets *AGO2*, thereby coordinating post-transcriptional gene silencing activities by different AGOs. Up-regulation of miR3627/4376 could facilitate anthocyanin accumulation by antagonizing a calcium effector, whereas miR395 and miR399, induced by micronutrient deficiencies known to trigger anthocyanin accumulation, respond positively to UV-B radiation. Finally, increases in the abundance of an anthocyanin-regulatory MYB-bHLH-WD40 complex elucidated in Arabidopsis, mediated by UV-B-induced changes in miR156/miR535, could contribute to the observed up-regulation of miR828. In turn, miR828 would regulate the *AtMYB113*-ortologues *MYBA5*, *A6* and *A7* (and thereby anthocyanins) via a widely conserved and previously validated auto-regulatory loop involving miR828 and phasi *TAS4abc* RNAs.

The sessile nature of plants makes them vulnerable to myriad abiotic and biotic stress factors to which they must adapt to survive. Ultraviolet (UV) irradiation, drought, heat, cold, salt, and oxygen deficiency are the major abiotic factors restricting plant growth, development, and productivity whereas microbial pathogens and herbivores are major biotic stress factors. Genome-wide association studies have identified UV-B responses with autoimmune-like reactions due to overexpression of defense related genes (Piofczyk *et al.* 2015). UV-B radiation (280-315 nm) is mostly absorbed by the ozone layer although about two percent passes through the atmosphere and causes detrimental effects on all life forms. A highly-conserved UV-B perception and signaling system is evolved in plants (Heijde and Ulm 2012; Tilbrook *et al.* 2016). Exposure to low-fluence doses of UV-B irradiation acts as an environmental trigger facilitating the expression of a wide array of genes involved in development or hormone signaling, including auxin, ethylene, abscisic acid (ABA), and cell wall modifying related genes (Frohnmeyer and Staiger 2003; Jenkins 2017; Mackerness 2000; Pontin *et al.* 2010; Ries *et al.* 2000). In addition to the up-regulation of antioxidant/free radical scavenging enzymes and proteins involved in DNA repair and cell cycle regulation (Brosché *et al.* 2002), a conserved and highly specific signaling pathway involving photomorphogenic receptors is also activated (Jenkins 2017). Furthermore, a few studies have shown that photomorphogenic responses to UV influence and/or are influenced by several miRNA activities. For instance, Cryptochrome1 and Cryptochrome2 blue light receptors mediate the expression of miR172 (Jung *et al.* 2007), whereas miR408 is coordinately regulated by SQUAMOSA PROMOTER BINDING PROTEIN-LIKE7 (SPL7) and ELONGATED HYPOCOTYL5 (HY5) transcription factors (TFs) in response to light (Zhang *et al.* 2014).

Plants fend off harmful effects of UV-B with phenylpropanoids (a group of plant-specific secondary metabolites, also known as polyphenols) derived from phenylalanine and deposited in the vacuoles of epidermal cells (Dotto and Casati 2017; Li, Ou-Lee *et al.* 1993). Flavonols, chalcones, and anthocyanins constitute a branch of metabolites from this pathway (known as flavonoids) that are synthesized by coordinated transcriptional control of a suite of biosynthetic enzymes, including *CHALCONE SYNTHASE* (*CHS*). The UV-B induction of *CHS* (Jenkins 2017) and the downstream flavonoid biosynthetic genes has been well characterized in several species. UV RESISTANCE LOCUS 8 (UVR8), a UV-B receptor, upon absorbing UV-B radiation monomerizes into its active form (Rizzini *et al.* 2011; Wu *et al.* 2012) and interacts with CONSTITUTIVELY PHOTOMORPHOGENIC1 (COP1) (Cloix *et al.* 2012). COP1 is a WD40/RING protein that facilitates proteasome-mediated degradation in the dark of HY5, a basic leucine zipper TF. COP1 interaction with UVR8 releases HY5 from proteasome-mediated degradation and promotes photomorphogenic signaling (Huang *et al.* 2013). Subsequently, HY5 binds to bZIP-binding promoter elements of *CHS*, *MYB12* (a regulator of flavonol biosynthesis; Lee *et al.* 2007, Stracke *et al.* 2010), and *MYB75/PRODUCTION OF ANTHOCYANIN PIGMENT1/PAP1* (a TF inducer of anthocyanin biosynthesis genes; Shin *et al.* 2013), increasing their transcription and facilitating the accumulation of flavonoids.

The grapevine (*Vitis vinifera* L.) is an important fruit crop abundant in health-promoting- and flavor-enhancing polyphenolics, with resilient metabolic and physiological responses to UV radiation. Despite the availability of its genome for over a decade, UV-B signaling components and their functions in integrated control of secondary metabolism have not been identified in this species until recently. Loyola *et al.* (2016) characterized the grape’s *UV-B RECEPTOR* (*UVR1*), *HY5* and *HY5 HOMOLOG* (*HYH*) that function in UV-B perception and signaling. The expression profile of the above-mentioned photomorphogenic factors was studied in vegetative and reproductive tissues at different developmental stages and in response to UV stress. While *UVR1* is strongly expressed in early berry developmental stages, transcript abundance is mostly influenced by light and temperature but not UV-B radiation. *HY5*, however, is induced by low-fluence UV-B in plantlets and at early fruit developmental stages (both with low- and high-fluence radiation expositions) (Loyola *et al.* 2016). *HYH* is induced by high-fluence UV-B three weeks after the onset of ripening, (veraison, manifested with the red/black pigmentation of berry skins and a transition from organic acid to sugar accumulation). UV-B irradiation also promoted flavonol accumulation in leaves and berries. Thus, both high and low intensity UV-B exposures in grapevine may facilitate flavonol accumulation in vegetative and reproductive organs through the activation of *HY5* and *HYH* at different developmental stages (reviewed by Matus 2016).

Anthocyanin accumulation is highly responsive to UV although is developmentally regulated by subspecialized R2R3-type MYB transcription factors. For example, in Arabidopsis SPL9, a target of miR156, negatively regulates anthocyanin accumulation during phase transition to flowering by directly preventing expression of anthocyanin biosynthetic genes through destabilization of a MYB/PAP1-bHLH/TT8-WD40 transcriptional activation complex (Gou *et al.* 2011). The grapevine berry color locus in chromosome 2 encodes the anthocyanin regulators *MYBA1* and *MYBA2* (Walker *et al.* 2007). Conversely, the red pigmentation observed in vegetative tissues is attributed to the activity of *MYBA6*, *MYBA7*, and potentially *MYBA5* genes, all clustered on chromosome 14 (Matus *et al.* 2017). *MYBA5/6/7* accumulate at high levels in early berry development and decrease after the onset of veraison. UV-B radiation induces *MYBA1* genes and significantly delays the down-regulation of *MYBA6* and *MYBA7* at the latter stages of berry development (Matus *et al.* 2017). Interestingly, *MYBA6* and *MYBA7* follow the exact expression pattern of grapevine *HY5* (Loyola *et al.* 2016), suggesting that *MYBA6* and *MYBA7* may also contribute to fruit pigmentation upon increased radiation stress, in addition to *MYBA1*.

Grapevine VviMYBA6 and VviMYBA7 are orthologs of AtPAP1/PAP2 and AtMYB113, the Arabidopsis MYBs known to be under negative regulatory control by the tasiRNA species TAS4-3′D4(-). These regulatory small RNAs are generated by miR828-mediated cleavage of the *Trans Acting Small-interfering RNA locus4 (TAS4)* (Rajagopalan *et al.* 2006). *AtMYB113* is targeted by miR828 directly in addition to *TAS4* 3′D4(-), suggesting a close evolutionary relationship between *AtPAP1*/*PAP2*/*MYB113*, miR828, and *TAS4* (Rajagopalan *et al.* 2006). *TAS4* is functionally conserved among various eudicots (Luo *et al.* 2012; Rock 2013) and in fact we have shown that TAS4-3′D4(-) targets *VviMYBA6* and *VviMYBA7*, triggering 21 nt phased small interfering RNA (phasi-RNAs) (Rock 2013; Zhang *et al.* 2012). The biological significance of 21 nt phasi-RNAs is largely unknown, but is hypothesized to be important for defining a concentration gradient for silencing activity across cell layers and for widespread *trans* suppression of large families, including Leucine Rich Repeat (LRR) resistance genes (Fei *et al.* 2013; Shivaprasad *et al.* 2012) or Pentatrico Peptide Repeat (PPR) genes (Howell *et al.* 2007).

Studies to date have focused on the influence of UV-B radiation in regulating anthocyanin biosynthesis at different developmental stages of berry development. The effect of UV-B on miRNA profiles is well-documented in *Arabidopsis*, poplar, maize and wheat (Zhou *et al.* 2007; Jia *et al.* 2009; Casati 2013; Wang *et al.* 2013), however the role of UV-B in post-transcriptional regulation of *MYB* TFs by miR828/*TAS4* pathway is unexplored. Further, there is a large annotation gap between the empirical knowledge of siRNA expression, especially the role of miRNAs and tasi-RNAs on production and functions of phasi-RNAs (Coruh *et al.* 2014; Luo *et al.* 2009). This work focuses on a data-driven systems approach to explore the effects of UV-B radiation on miRNA/phasi-RNA expression and elucidate functional consequences for stress adaptation by controlling the biosynthesis of UV-B absorbing anti-oxidant secondary metabolites. We performed a detailed computational re-analysis of empirically characterized grapevine miRNAs for slicing activities with a degradome library (Pantaleo *et al.*, 2010). We then performed quantitative real-time PCR (qRT-PCR) assays on miRNA target mRNAs in same-source samples used to generate our small RNA (sRNA) libraries and obtained supporting evidence for a regulatory network model of UV responses mediated by sRNAs. Meta-analysis of available mRNA transcriptome results for similar experiments across UV treatments and developmental time points (Carbonell-Bejerano *et al.* 2014; Massonnet *et al.*, 2017; Suzuki *et al.* 2015) further corroborate the model for sRNAs fine-tuning abiotic and biotic stress responses.

## Materials and Methods

### UV-B treated in vitro plantlets and fruits

In order to gain perspective in defining berry-specific relationships (Loyola *et al.* 2016; Czemmel *et al.* 2017) relative to leaf UV-B (Cavallini *et al.* 2015) sRNA responses, *in vitro* grown plantlets (four negative control and four UV-B treated biological replicates; n = 4) from the cultivar (cv.) ‘Cabernet Sauvignon’ were exposed to 6 hr of low-fluence UV-B radiation (∼0.15 W m^-2^ irradiance) exactly as conducted in Cavallini *et al.* (2015). Leaves were harvested after +UV-B treatment and frozen in liquid nitrogen for RNA extraction. Control plants were grown under the same conditions described above but covered with a polyester filter (100-μm clear safety polyester plastic film) that absorbed total UV-B from the spectrum without affecting the photosynthetic active radiation.

Potted nine-year-old greenhouse-grown cv. ‘Cabernet Sauvignon’ fruit clusters were exposed to high (∼0.3 W m^-2^ daily for 5 hr) UV-B or low (∼0.1 W m^-2^ daily for 10 hr) UV-B irradiance during 2011-2012 and 2012-2013 growing season, respectively (both contrasted to control ‘filtered UV-B’ conditions; more details in Loyola *et al.* 2016). Sample collection was carried out at four stages of grapevine development: -3, 0, 3 and 6 weeks before (-) and after (+) veraison (WAV, defined as the time at which clusters are 30–50% colored and sugar concentration reaches 5° Brix). Berries were immediately peeled, deseeded and skins were frozen in liquid nitrogen for RNA extraction. A total of 18 berries were sampled every 3 weeks from 9 grape clusters (n = 3, three bunches per plant and two berries per cluster). One and three biological replicates were considered for deep sequencing and qRT-PCR analyses, respectively.

A solar UV-B filtering treatment was applied in a commercial cv. ‘Cabernet Sauvignon’ field, located in the Maipo Valley (Chile; 33◦ 43′ 28′′ S, 70◦ 45′ 9.71′′ W) during 2011-2012 growing season exactly as conducted in Czemmel *et al.* (2017). Berry skin samples were collected at -3, 0, +3 and +6 WAV from a row of UV-B filtered (UV-B radiation was blocked by installing a 100-μm clear polyester film at the position of grape clusters) and +UV-B irradiated plants (natural solar radiation, no filter). The experiment was designed in four blocks (rows) with five plants each (biological replicates *n* = 4). Three berries per cluster (randomly sampled) and four clusters per plant were used for each sample. One biological replicate was considered for deep sequencing.

In total, 28 samples were used for sRNA library construction; four for *in vitro* plantlets (two biological replicates, two conditions), 16 for greenhouse-grown berries (one biological replicate, four-time points, four conditions [with and without high- or low-fluence UV-B), and eight for field-grown berries (one biological replicate, four time points, two conditions [with or without solar UV-B]).

### RNA extraction and sRNA libraries construction

Total RNA was isolated following the procedure of Reid *et al.* (2006) using a CTAB- spermidine extraction buffer. The RNA was quantified using NanoDrop 1000 spectrometer (Thermo Fisher Scientific). The integrity of RNA was assessed by an Agilent 2100 Bioanalyzer using a sRNA chip (Agilent Technologies) according to the manufacturer’s instructions.

sRNA libraries were prepared using total RNA as input (1 µg) according to the instructions provided by TruSeq sRNA Sample Preparation Kit (Illumina). Twenty-eight bar-coded sRNA libraries were constructed. The quality of each library was assessed using an Agilent High Sensitivity DNA chip on an Agilent 2100 Bioanalyzer. Equi-molar concentrations of libraries were pooled and sequenced on an Illumina NextSeq500v2 instrument at the Institute for Integrative Genome Biology, UC Riverside. One library (minus UV-B, -3 WAV, high-fluence UV greenhouse experiment) was re-sequenced to increase reads depth on a MiSeqv3 platform, and another library was solely sequenced on MiSeqv3 (plus UV-B, +6 WAV, field grown; Supplementary Table 1a). This latter library had consequently fewer reads and the 21 and 24 nt species of small RNAs were not as abundant due to overrepresentation of a specific 48 nt non-coding RNA homologous mapping to chromosome 11 intergenic region.

### Bioinformatics and Statistical Analyses of Sequencing Data

The quality assessment of the raw reads was done by FastQCv0.11.5 (https://www.bioinformatics.babraham.ac.uk/projects/fastqc/) and adaptor sequences were trimmed using fastx_clipper (http://hannonlab.cshl.edu/fastx_toolkit/index.html). Reads longer than 18 nt were retained and were sequentially mapped to *Arabidopsis thaliana* rRNAs, tRNAs, snRNAs, and transposable elements (TEs; downloaded from https://plants.ensembl.org/info/website/ftp/index.html and www.arabidopsis.org) using Bowtie (Langmead *et al.* 2009) to remove reads matching the same. Bowtie was used to map RNAseq datasets of Massonnet *et al.* (2017; NCBI BioProjects PRJNA265039 and PRJNA265040) to pre-*MIR828* and downstream effector targets quantified by qRT-PCR (see below) in our field samples. There remained a few abundant 32-53 nt long reads in the libraries after pre-filtering that mapped to XR_002032162.1 “Vitis uncharacterized ncRNA”, XM_002283070.4 “DELETION OF SUV3 SUPPRESSOR 1”, a snoRNA, and a 5.8S rRNA. The pre-filtered reads were mapped to the reference *Vitis vinifera* 12X genome sequence, version NCBI RefSeq GCF_000003745.3 (https://www.ncbi.nlm.nih.gov/assembly/GCF_000003745.3) (Jaillon *et al.* 2007) using ShortStack (version 3.8.2) (Johnson *et al.* 2016). In order to increase the power of ShortStack to call *MIRNA*s by sequencing at least one rare miRNA star species, we included publicly available sRNA datasets for grapevine from the following sources available from NCBI: Belli Kullan *et al.* (2015; SRR1528350- SRR1528419), Paim Pinto *et al.* (2016; SRR4031591- SRR4031638), Pantaleo *et al.* (2010; 2016; GSM458927- GSM458930; GSM1544381- GSM1544384), Sun *et al.* (2015; SRR2029783, SRR2029784), Wang *et al.* (2012, 2014; SRR390296, GSM1907875- GSM1907880), and Zhai *et al.* (2011; GSM803800- GSM803802). The ShortStack counts output for these libraries were not used for subsequent differential expression analysis. ShortStack counts output generated from our UV treatment libraries alone were subjected to differential expression analysis.

A few novel candidate *MIRNA*s were called *de novo* by ShortStack as having met all annotation requirements (*viz*., > 50% of reads map to hairpin duplex, >80% of reads map to one strand of the hairpin, and at least one miRNA star species sequenced). The raw counts output of ShortStack for 40,678 sRNA-producing loci with greater than 261 total reads per cluster summed across all libraries (equivalent to ∼0.5 rpm raw reads, the default for ShortStack cluster calling) was used as input to DESeq2 “R” Package (version 1.16.1) (Love *et al.* 2014) to analyze the differential expression of sRNA cluster loci under different conditions. The cutoff was chosen to capture the low-expressed (∼1.5 reads per million, rpm) *MIR828* and *MYBA6* and *MYBA7* clusters, which were of particular interest but at the cost of more independent statistical tests. For comprehensive genome-wide assessment, an additional 25 clusters mapping to miRBase21-annotated grapevine *MIRNAs* (Kozomara *et al.* 2018) were included which were below the cutoff of 261 total reads. This preprocessing step of limiting candidate clusters increases the power of DESeq by removing poorly expressed loci that would otherwise contribute to ‘shot noise’ variation and excessive multiple test corrections for statistical significance, while adequately encompassing nearly all annotated *MIRNA* loci (*e.g.*, *MIR858*, 0.4 rpm; *MIR395f*, 0.05 rpm (see Supplementary Table 3).

The experimental comparisons made for differential expression analyses of biologically coordinated replicates are outlined in Table 1. The differential expression of *MIRNA* loci and phasi-RNA-producing loci as a time-series spanning berry development was tested using the likelihood ratio test (LRT) for inference, which assumes that the expression data follows negative binomial general linear model. To detect differentially expressed genes under +UV/-UV and high/low fluence UV radiation, Wald-Log test was performed. Publicly available grape degradome data (Pantaleo *et al.* 2010; GSM458931) and the sRNA library datasets were subjected to PhaseTank analysis (Guo, Qu, & Jin 2015) to identify the genome-wide phased siRNA-producing loci and regulatory cascades triggered by miRNAs. PhaseTank is built on CleaveLandv4.4.3 (Addo-Quaye *et al.* 2009). CleaveLand4 is more sensitive than earlier versions employed in prior analyses of GSM458931 (Pantaleo *et al.* 2010) by implementation of Generic Small RNA-Transcriptome Aligner (GSTAr) which calculates duplex parameters on RNA-RNA thermodynamics instead of sequence-based alignment. For False-Discovery Rate correction, the genome-wide numbers of *MIRNA* loci and phasi-producing loci identified by ShortStack and PhaseTank, respectively, were used as the basis for independent tests.

**Table 1:** Schematic of various differential expression comparisons described in Results

Experiment	Comparisons	DESeq2 Statistical test
Supplementary Table S3a. Effect of low-fluence UV-B on *in vitro* plantlets	+UV-B plantlets **vs** -UV-B plantlets. Two biological replicates	Wald-Log test
Supplementary Table S3b. Effect of UV-B on berries	+UV-B (low-fluence, high-fluence and field samples) **vs** coordinate -UV-B (low-fluence, high-fluence and field samples). Three replicates	Wald-Log test
Supplementary Table S3c. Effect of UV-B fluence on berries	+UV-B and -UV-B high-fluence **vs** co-ordinate +UV-B and -UV-B low-fluence. Four replicates	Wald-Log test
Supplementary Table S3d. sRNAs differentially expressed during berry development	−3 WAV, 0, and +3 **vs** +6 WAV coordinated to three conditions (low-fluence, high-fluence and field samples) and two treatments (+UV, -UV). Three replicates	Likelihood Ratio Test

### Identification of conserved miRNAs and targets

To identify the conserved miRNAs, all miRNA candidates and sRNA clusters called by ShortStack were annotated to genome coordinates of 163 vvi-miRNAs deposited in miRBase (version 21; Kozomara *et al.* 2018; http://www.mirbase.org/). To identify the mRNA targets that are sliced by miRNAs, CleaveLand 4.4 (Addo-Quaye *et al.* 2009) analysis was performed. Four degradome datasets were used for this analysis. Publicly available grape degradome data (Pantaleo *et al.* 2010; GSM458931) was pre-filtered through *Vitis vinifera* ncRNAs in Rfam (Kalvari *et al.* 2017) (Suppl. Table. 1b) and was used as the *bona fide* degradome input. We also created three independent “pseudo-degradome” libraries by concatenating small RNA libraries. The rationale for using small RNA libraries as pseudo-degradome inputs for CleaveLand analyses is grounded in our interest to study those 5′-monophosphate sliced polyadenylated transcripts, which are templates for production of phasiRNAs triggered by miRNA activities. Such species of 21 nt diced dsRNA products are well-represented in sRNA libraries (Luo *et al.*, 2009; Rock 2013). Further, independent corroboration of slicing activities from different sources improves the statistically defensible rigor of CleaveLand (Addo-Quaye *et al.* 2009). We therefore concatenated all 28 UV-B test sRNA libraries and employed them as a biological replicate, as well as the four sRNA libraries from Pantaleo *et al.* (2010) and independently the 14 sRNA datasets used for *MIRNA* empirical characterizations with ShortStack (Belli Kullan *et al.* 2015; Paim Pinto *et al.* 2016; Sun *et al.* 2015; Wang *et al.* 2012, 2014; and Zhai *et al.* 2011). The identification of most of the canonical, deeply conserved miRNA targets validated the robustness of the approach. For example, Pantaleo *et al.* (2010) identified targets for 13 conserved miRNA families out of 24, but not miR168, miR395, miR396 and miR403 despite high effector abundances. We validated 126 targets from 27 of 31 deeply conserved miRNA families, including for miR168, -395, -396, and -403 (Supplementary Table 2a and Supplementary File S4). When a known canonical miRNA target was not validated due to absence of slice signature reads in degradome datasets (miR397, -399, -408, and -827), we relied on Generic Small RNA-Transcriptome Aligner (GSTAr) output with low Allen scores to identify the missing canonical predicted targets (all found except for miR827 targets; Supplementary Table 2a). It is noted that vvi-miR397 target *LACCASE11* (Supplementary Table 2a) has been previously validated by 5′ RNA Ligase-Mediated Rapid Amplification of cDNA Ends (RLM-RACE) in grapevine (Sun *et al.* 2015). We further obtained evidence for novel targets of some conserved miRNAs (miR169, -395, -396, -477, -530, -535, and -828) and for grapevine-specific miRNAs miR3623, -3624, -3626, -3629, -3631, -3632, 3633, and miR3635 (Supplementary Table 2a, Supplementary File S4). The *JAZ4* homolog of miR169 novel target *JAZ3_1* transcription corepressor (Supplementary File S4, p. 14) has recently been demonstrated along with canonical miR169 targets *NF-YA*s to be an important effector of temperature adaptation and phenology in Arabidopsis by interaction with APETELA2-like TARGET OF EAT1/2 (targets of miR172; Supplementary File S4, p. 17; Supplementary Table 2a) that negatively regulate FLOWERING TIME (Gyula *et al.* 2018). The miRNA targets were also predicted in parallel using the psRNATarget: A Plant sRNA Target Analysis Server (Dai *et al.* 2018) and Plant Non-coding RNA Database (PNRD) (Yi *et al.* 2015). The expectation threshold of psRNATarget was set to an Allen score = 4 (Fahlgren and Carrington 2010) for target predictions (Supplementary Table 2b).

ShortStack output (Supplementary Table 3 columns Q and S) lists the “Major RNA” (*i.e.*, most abundant) species and “Complexity”, calculated as (n distinct alignments)/(abundance of alignments). Low complexity values indicate loci dominated by just a few sRNAs. Although the ShortStack metrics as reported were the average across all libraries, the Major Species reported is invariably the mature miRNA species (or star, when it is claimed as functional, as with miR3624-3p). Further, the Complexity reported is quite low such that inference can be made that differentially expressed sRNAs are predictable. The average Complexity was 0.03 for claimed *MIRNA* clusters (data not shown), with several loci having zero Complexity (see Supplementary File S6 for documentation of complexity vis-à-vis reads abundances for novel *MIRNA*s). Complexity averaged 0.07 for *PHASI* loci across all claimed loci. For *TAS4a*, where D1(+) was the major species, the Complexity was 0.002 and our 28 libraries accounted for ∼11% of all mapped *TAS4a* reads (data not shown); for *TAS4b* the functional 3′-D4(-) siRNA was the major species and Complexity was 0.001 wherein our libraries accounted for 1% of all T*AS4b* reads *vs.* the public data used for annotation power. For *TAS4c*, 3′-D4(-) was the major species and the measured Complexity was 0.01; for this key hypothesized effector of *MYBA6* and *MYBA7* expression our libraries accounted for 91% of all *TAS4c* cluster reads. Taken together, these data support our view that statistical inference of miRNA and siRNA dynamics using clusters as proxy for *bona fide* effectors is reasonable and useful.

### RNA blotting and Densitometry

Total RNA (15 µg) was loaded onto a 17% polyacrylamide gel with 7M urea and was electrophoresed in 0.5X TBE at 200 V. RNA was blotted onto the positively charged Amersham Hybond-N^+^ nylon membrane (GE Healthcare Life Sciences, USA) using the Owl semidry transfer apparatus (Thermoscientific, USA) and the membrane was UV-crosslinked (SpectroLinker XL-1500, Spectroline, Westbury NY). A 22-nt anti-vvi-miR828 Locked-Nucleic Acid oligonucleotide (Válóczi *et al.* 2004) (Exiqon Inc., Woburn, MA; www.exiqon.com) was end labeled with [γ-^32^P]ATP, 6,000 Ci/mmol, (Perkin Elmer, www.perkinelmer.com) and used as probe. Hybridization was performed at 37° for 16-20 h with PerfectHyb Plus hybridization buffer (Sigma). Post-hybridization washes were performed as follows: The hybridization solution was discarded, and the blots were washed sequentially with 2X SSC/0.2% SDS, 0.5X SSC/0.2% SDS and 0.1X SSC/0.2% SDS. Each wash was done for 30 min at 37° in the hybridization oven. As an equal loading control, the membrane blot was re-probed with end labeled [γ-^32^P]ATP 5S rRNA oligonucleotide. RNA blots were scanned using a Storm 860 PhosphorImager and sRNA signals were quantified using ImageLab software (v6.0, Bio-Rad. www.bio-rad.com). We segmented the miR828, 5S rRNA band areas into seven vertical subsections of equal area per lane. Values of five subsections were averaged after discarding the leftmost and rightmost subsections, which in some lanes showed gel migration artifacts. An area of the same subsection dimensions adjacent to bands was subtracted from the signal to remove background noise. A ratio of miR828 average signal divided by the control 5S RNA average signal from each lane was calculated to normalize across samples for lane loading variations. The test comparison (UV treatment -fold change) for expression strength was calculated as the ratio of normalized plus UV sample signal divided by the cognate minus-UV sample normalized signal (set to unity) for a given tissue (*in vitro* plantlet replicates), developmental time point, greenhouse fluence condition, and field condition.

### Quantitative Real-Time PCR

cDNA amplification was conducted as in Matus *et al.* (2009). Quantification of relative gene expression was carried out as in Dauelsberg *et al.* (2011), following a thermocycling program of 95° for 10 min followed by 40 cycles at 95° for 20 s, 55-60° for 20 s, and 72° for 20s, followed by 71 cycles increasing from 50 to 96° at increments of 0.5° per cycle for 30s to obtain a melting curve. Standard quantification curves with serial dilutions of PCR products were constructed for each gene to calculate amplification efficiency. Melting temperatures, primer sequences and amplification efficiency coefficients are shown in Supplementary File S7. *VviUBIQUITIN1* was used as reference gene for calibration. To differentiate the transcripts of *Tas4a*, *Tas4b* and *Tas4c* and to amplify specific transcripts, primers were designed flanking the endonucleolytic slicing sites for miR828 and TAS4 3′D4(-) in highly variable regions (Supplementary Fig. S2A). qRT-PCR amplicons were run in 1% agarose gels by electrophoresis and purified and sequenced to validate appropriate designs. All experiments were performed with three (greenhouse experiment) and four (field experiment) biological replicates and two or three technical replicates.

### Data availability

The raw data files of UV sample libraries can be accessed from the National Center for Biotechnology Information (NCBI) under Sequence Read Archive accession SRP131396. Supplemental material available at Figshare: https://doi.org/10.25387/g3.7471553.

## Results

**Figure 1:**
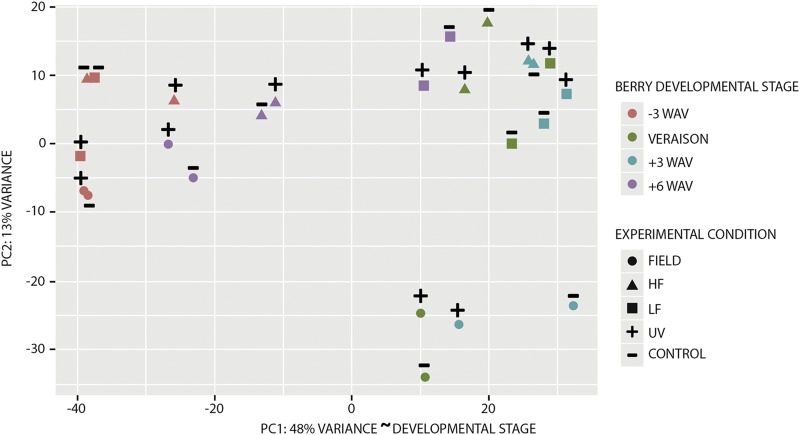
Principal component (PC) analysis of all 24 grape berry library sRNA-generating loci subjected to differential expression analysis. The percentage of variation is depicted in the PC1 and PC2 axes. Based on temporal clustering of colors, PC1 is inferred to capture the developmental stage (WAV, weeks after veraison) of samples. HF: high-fluence UV-B greenhouse berries; LF: low-fluence UV-B greenhouse berries.

As UV-B irradiation increases the expression of *VviHY5* and of its regulatory network (*i.e.*, *MYBF1*, *FLS4*, *MYBA6* and *MYBA7*) in both leaves and berries of UV-treated plants, favoring flavonol and anthocyanin accumulation (Loyola *et al.* 2016; Matus *et al.* 2017; Czemmel *et al.*, 2017), we choose to explore sRNA abundances in all these previous conditions in order to provide a perspective in defining organ-specific UV-B responses. Small RNA (sRNA) libraries were constructed for four *in vitro*-grown leaf samples (control and UV-B treated, two biological replicates) and 24 berry skin samples (control and UV-B irradiated/filtered samples collected from greenhouse and fields at -3, 0, +3 and +6 Weeks After Veraison, WAV) and sequenced by Illumina NextSeq500v2 (1 × 75 × 6 cycles for indexes). We obtained a total of 356,090,480 raw reads (Supplementary Table 1a). After adaptor trimming and removal of rRNA, tRNA, snRNA, and TE-like sequences longer than 18 nt found in the 28 libraries (Supplementary Table 1a), there remained 231,853,790 clean reads (65.7% of raw reads), of which 8% on average were unique within libraries and 5.2% were unique across all libraries. Reads longer than 27 nt were retained in the dataset for quality control assessment of libraries, which contained discreet fragments of grapevine mRNAs and a few ncRNAs, snoRNA and rRNA species of 32, 48, 51, and 53 nt sizes not filtered with Arabidopsis sequences (Supplementary Fig. S1). ShortStack mapped 31% of reads uniquely to the reference genome, while 32.8% of reads mapped to multiple loci and were binned proportionally based on flanking sequence uniqueness (Johnson *et al.* 2016). Only 0.3% of the reads mapped to more than 50 loci and were discarded. Principal component analysis of berry samples showed a near majority of variation in abundances of sRNAs mapping to 40,678 loci between libraries that was strongly associated with berry development (48%, PC1) (Figure 1). The chronological sequence from green berry (-3 WAV), veraison, +3 WAV, and +6 WAV clustered coordinately and continuously irrespective of samples being collected from greenhouse or field (Figure 1). The +6 WAV sRNA genome-wide variations were more dynamic (clustered loosely) than earlier well-staged/constrained time points, consistent with greater transcriptome variation at harvest reported by Massonnet *et al.* (2017) among red berry cultivars than between red and white cultivars.

The size distributions of trimmed reads were analyzed showing that all libraries had distinct peak abundances at 21- and 24-nt (Supplementary Fig. S1). Consistent with previous reports in grapevine (Pantaleo *et al.* 2010; Paim Pinto *et al.* 2016), the 21-nt species was more abundant when compared to the 24-nt sRNA species. To identify the conserved miRNAs, all miRNA candidates and sRNA clusters called by ShortStack were annotated to genome coordinates deposited in miRBase. The following *MIRNA*s were not found in the ShortStack dataset: vvi-*MIR169akw*, -171dgj, -395k, -399f, -535a, -845ade, and vvi-*MIR3631c*. Other *MIRNA*s identified in the dataset and reported in the literature but not in miRBase were vvi-miR529, vvi-miR530 (Pantaleo *et al.* 2010), vvi-miR827 (Alabi *et al.* 2012; Han *et al.* 2016; Pantaleo *et al.* 2010), vvi-miR858 (Pantaleo *et al.* 2010), vvi-miR4376, vvi-miR5225 (Wang *et al.* 2011; Xia *et al.* 2013), and vvi-miR7122 (Pantaleo *et al.* 2016; Xia *et al.* 2013). Also present in the dataset as differentially regulated we identified 13 novel candidate *MIRNA*s, including several with weak hairpin homology to other plant species in the miRBase, *e.g.*, miR5741/8685, miR7099, and miR8761. The novel *MIRNA* hairpin sequences, their refseq genome coordinates, and read counts are documented in Supplementary File S6. The miRNA expression profiles under different experimental set up and their experimentally confirmed/putative mRNA targets are discussed in detail below.

### UV-B responsive miRNAs in in vitro-grown plantlets

Two biological replicates from low-fluence UV-B irradiated plants and two samples from the control treatment were analyzed for differential expression of miRNAs (Supplementary Table 3a). Two UV-B responsive miRNAs, vvi-MIR5225 and vvi-MIR160e, which target the phasi-RNA spawning locus *Ca^2+^-ATPase10* (Supplementary Table 2a) and ARF10/17 TFs, respectively (Supplementary Table 2a; 2b; Supplementary File S4, p5), were identified as significantly up-regulated (Supplementary Table 3a). miR5225 is a 22 nt species evolutionarily related to miR3627 and miR4376 (Xia *et al.* 2013; Wang *et al.* 2011). In grape miR5225, miR3627 and miR4376 target the 5′UTR intron2 region of the calcium-ATPase10, 14-40 nt upstream of the translation start site as independently demonstrated by the CleaveLand analyses of miR4376 and iso-miR3627 in public and Pantaleo *et al.* (2010) sRNA pseudo-degradome datasets (Supplementary Table 2a; Supplementary File. S4, pp. 56, 57). The orthologous genes in cassava, hop, and mangrove have expressed sequence tags (NCBI JG970650.1, ES653410.1, DB992577.1) mapping to the miR5225/3627/4376 target site and downstream through the translation start site, suggesting *calcium-ATPase10* intron2 is mis-annotated.

Although vvi-MIR482 expression was not significantly changed by UV treatment in *in vitro* plantlets (*P* = 0.21, possibly due to small sample size and high read number), PhaseTank analysis revealed a non-coding phasi-RNA candidate *TAS* locus (chr13:15496204-15497757) (ShortStack phase score 1,737) generating a tasiRNA [3′D3(-)] by vvi-miR482 cleavage (Allen score = 5.0; *P* < 0.07) (Supplementary Table 4 and File S5). This novel candidate *TAS* locus significantly up-regulated by UV treatment (Supplementary Table 3a) was named vvi-*TAS11* following the nomenclature of Zhang *et al.* (2012). vvi-*TAS11* maps to the 5′ end of an unannotated 87aa peptide-encoding locus *VIT_13s0047g00100* with ESTs (GenBank XR_002031633.1, XR_787251.2) annotated as ’uncharacterized non-coding RNA’. Additionally, degradome evidence (Allen score 4.0; Supplementary Table 4) supports cleavage of a phasi-RNA-producing (phase score =93.5) Leucine-Rich-Repeat (LRR) transcript by the tasiRNA3′-D3(-), which is predicted to target another phasi-RNA-producing LRR (phase score 29.2), 12 NB-ARC-LRRs (Supplementary Tables 2b, 3a, 4), and the putative cation/hydrogen exchanger *Vvi-CHX15*.

**Figure 2:**
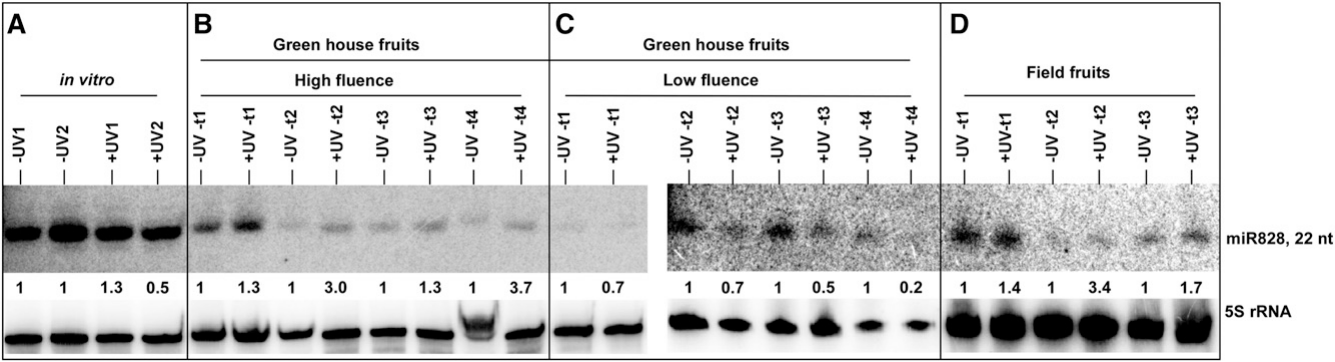
sRNA blot of miR828 abundance in UV response samples subjected to deep sequencing. The quantitation of mature miR828 abundances by densitometry was used to validate ShortStack quantitation of relative vvi-*MIR828* sRNA cluster abundances, which are used in statistical analyses as a proxy for mature miR828 species abundance since mostly miR828* species were sequenced from sRNA libraries. A) UV-induced expression of miR828 in *in vitro* plantlets. –UV: No UV-B treatment; +UV-B: 6 hr of low UV-B radiation (0.15 W m^-2^ irradiance). B) UV-induced expression of miR828 at different stages of berry development in greenhouse conditions subjected to high-fluence UV-B. –UV: No UV-B treatment; +UV-B: (∼0.3 W m^-2^ daily for 5 hr). C) UV-induced expression of miR828 at different stages of berry development in green house conditions when subjected to low-fluence UV-B. –UV: No UV-B treatment; +UV-B: (∼0.1 W m^-2^ daily for 10 hr). D) UV-induced expression of miR828 at different stages of berry development in field conditions UV-B. –UV: Solar UV-B blocked by 100 μm clear polyester film; +UV-B: No filters. Panels B-D: t1, t2, t3 and t4 corresponds to Weeks -3, 0, 3, and 6 after veraison (WAV), respectively. As loading control, 5S rRNA probe was hybridized to the same membrane. The relative abundance of miR828 in test samples (+UV-B) is presented as the ratio compared to normalized abundance of -UV-B controls (set to unity).

Induction of *MYBA6* and *MYBA7* in vegetative tissues upon UV-B radiation has been observed previously (Matus *et al.* 2017). Similarly, miR828/*TAS4*-mediated post-transcriptional regulation of *MYBA6* and *MYBA7* by production of TAS"4-3′D4(-)-triggered phasi-RNAs is also well documented in leaves (Rock 2013). The extremely low levels of mature miR828 (∼1 per 20 million reads) in the sRNA libraries, including leaves (where it is expected to be maximal), made it difficult to quantify by deep sequencing. Hence, a more sensitive RNA blot analysis with Locked Nucleic Acid-anti-vvi-miR828 as probe was performed to verify the expression profile of *MIR828* cluster siRNAs (miR828* was the major species found at 2.6 reads per million; Supplementary Table 3c,d) quantified by ShortStack. As predicted, mature miR828 was maximal in leaves, but there was no obvious difference in miR828 levels in response to UV-B in plantlets (Figure 2; Panel A; but see below).

### UV-B responsive small RNAs in berry skins from greenhouse and field experiments

The high- and low-fluence UV irradiated greenhouse and field samples were classed as +UV-B test samples and the corresponding minus (-) UV-B as the control samples for differential expression analysis (Table 1). Eleven UV-B responsive miRNAs were identified with statistically significant differential expression (independent of developmental time points), of which nine were up-regulated (vvi-MIR395n, vvi-MIR3627, vvi-MIR535c, vvi-MIR3624, vvi-MIR171b, vvi-MIR156f, vvi-miR4376, vvi-MIR319c) and two were overall down-regulated (vvi-MIR477b, vvi-MIR530) (Supplementary Table 3b). Conversely, further analysis (described below) showed those two down-regulated *MIRNA* loci are up-regulated in response to high-fluence UV-B. The up-regulated UV-B-responsive miRNA targets were validated by CleaveLand analysis and corroborated by psRNATarget, Plant Non-coding RNA Database (PNRD) (Yi *et al.* 2015), and evidences from available literature (see Supplementary Table 3b; Supplementary Tables 2a, 2b; Supplementary File S4). CleaveLand analysis validated *Metal Ion Binding Protein* mRNA, previously reported by Sun *et al.* (2015) and Pagliarani *et al.* (2017) as the target of MIR3624-3p (Supplementary Table 2a; Supplementary File S4, p. 62), despite the *MIR3624* locus produces abundant phasi-RNAs from the antisense strand (Supplementary Table 3b).

CleaveLand and PhaseTank analysis identified vvi-MIR482 targeting a second *TAS* locus (chr18: 20665102-20666018), annotated as overlapping the 3′ UTR of an 83aa transmembrane peptide transcript *VIT_18s0072g01090* (Supplementary Table 2a, 2b; Supplementary File S4, p. 37). An abundant tasiRNA 3′D12(-) significantly up-regulated by UV is analogous by concordance of phase (but not by homology) to sly-*TAS5* (Li *et al.* 2012; data not shown) and likewise is predicted to target eight NB-ARC/TIR-LRR transcripts (Supplementary Table 2b, File S5), thus we named it vvi-sly*TAS5*-like. This candidate *TAS* locus produces a tasiRNA3′-D6(+) predicted to target NB-LRRs (Supplementary Table 4 PhaseTank prediction, Supplementary File S5). Degradome evidence demonstrated the tasi-RNA D6(+) from vvi-sly*TAS5*-like also directs cleavage (Allen score 3.5) of an Armadillo-type fold domain found in nuclear cap-binding protein CBP80 and BB-LRR variants an S-adenosyl-methionine-dependent methyltransferase in the family of catechol/caffeoyl-CoA phenylpropanoid biosynthesis enzymes. Another PHASI locus with an unknown trigger produced an abundant siRNA up-regulated by UV-B with homology to miR6173 (Liu *et al.* 2015; data not shown). We identified from degradome analysis a phasi-RNA-producing and *cis*-cleavage active locus triggered by miR390 possibly involving the 2_21_ two-hit mechanism (Axtell *et al.* 2006; Supplementary Table 2a; Supplementary File S4, p. 18; Supplementary File S5) associated with AGO7 complex (Montgomery *et al.* 2008; Rajeswaran and Pooggin 2012). These phasi-RNAs are generated from *VIT_12s0059g01410* encoding a 66aa signal peptide with limited homology to Arabidopsis *TASIR-ARF/At5g57735* and distinct from *VviTAS3* triggered by miR390 (Zhang *et al.* 2012; Supplementary File S4, p. 19; Supplementary File S5, see below). In addition, a phasi-RNA-producing locus (unannotated, but homologous to rice Oryza|ChrUn.fgenesh.mRNA.47_GX23P; https://phytozome.jgi.doe.gov) was predicted to be targeted by miR2111 (Supplementary Table 4, prediction sheet), as well as some evidence for miR2111 slicing of *NHD1*, Sodium hydrogen antiporter (Supplementary File S4, p. 58), but miR2111 was not significantly regulated by UV-B and not considered further.

Matus *et al.* (2017) reported that berries irradiated with UV-B delayed the decline in *MYBA5/6/7* expression after the onset of veraison, supporting the role of *MYBA6* and *MYBA7* in favoring pigmentation during fruit development under increased abiotic stress conditions. Luo *et al.* (2012) and Hsieh *et al.* (2009) characterized in Arabidopsis an auto-regulatory feedback loop involving the up-regulation of miR828/*TAS4* by AtPAP1/MYB75 (an ortholog of *VviMYBA6/7)* and being themselves targets of the negative regulators miR828/*TAS4*. Piya *et al.* (2017) recently described a similar auto-regulatory loop for miR858 and *MYB83*. If such feedback loops are conserved in grapevine, an increase in *MYBA6/7* transcript levels would increase the levels of miR828- and/or miR828-triggered *TAS4*-3′-D4(-) from *VviTAS4abc* loci, which cleave *MYBA6/7* to generate phasi-RNAs that in turn amplify the post-transcriptional silencing of these *MYBA* genes (Rock 2013). Inference of miR828 expression by deep sequencing read abundances of miR828* (being much more abundant than the rarely sequenced miR828), suggested overall down-regulation of *MIR828* locus by UV-B treatment (Supplementary Table 3b). Yet we observed a significant UV-B induced accumulation of *TAS4b*-3′D4(-) (Supplementary Table 3b ^ footnote) that is functionally contingent upon miR828 activity, consistent with the results of Matus *et al.* (2017) and the hypothesized auto-regulation of miR828/Vv*TAS4abc* loci. This hypothesis was substantiated by CleaveLand analysis of sRNA pseudo-degradome in which we observed miR828 slicing of *VviTAS4a/b/c* and the phased *TAS4ab*-3′D4(-) species targeting *MYBA7*, *MYBA6*, and *MYBA5/MYB113-like/VIT_14s0006g01340* (Supplementary Table 2a; File S4, pp. 38-43). Because vvi-*MIR828* was not significantly correlated to UV-B by DESeq2 analysis across all fluence and field treatments, we characterized mature miR828 expression alone by RNA blot (Figure 2). Interestingly, semi-quantitative densitometry analysis of band intensities showed reproducible up-regulation of miR828 by high-fluence UV-B treatments in both greenhouse and field fruits, and down-regulation of miR828 by low-fluence UV-B greenhouse treatments (Figure 2B-D). The binomial distribution probability for the concordant UV-B up- and down-regulations of mature miR828 across all 11quantified greenhouse and field sample signals of Figure 2 to occur by chance was *P* < 0.0005, demonstrating a robust UV-B response of mature miR828 despite the very low abundance found in our sRNA libraries. These results suggest a complex regulation (up-regulation by high-fluence despite being down-regulated by low-fluence UV-B) of vvi*-MIR828* locus, representing a behavior possibly shared by other miRNAs. miR828 also appeared to be down-regulated over developmental time in high-fluence UV-B experiments (Figure 2B, D).

**Figure 3:**
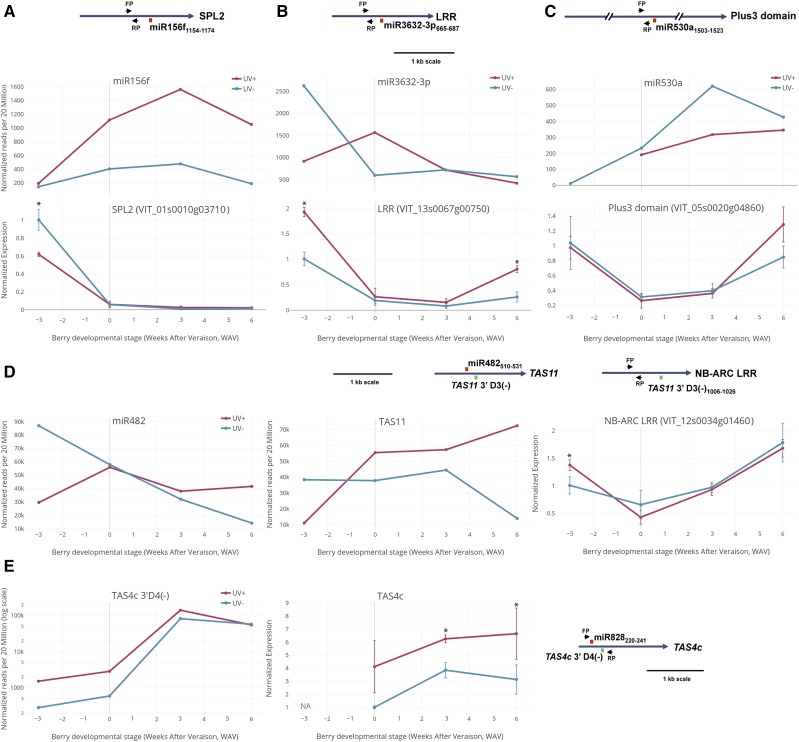
Functional validation of differentially expressed miRNA activities under high-fluence UV-B by qRT-PCR of target genes. Upper Panel A-C, left panel D: miRNA/siRNA deep-sequencing profiles at different berry developmental stages (weeks after veraison, WAV) under high-fluence UV-B in greenhouse. Lower Panels A-C, right panel D: miRNA target gene expression profile at different berry developmental stages under high-fluence UV-B. A) miR156f and its target Squamosa promoter binding protein-like 2 (SPL2) expression profile. B) miR3632-3p and its target Leucine-rich repeat protein (LRR) expression profile. C) miR530a and its target Plus3 domain protein expression profile. D) miR482 expression profile (left panel). Middle Panel: *TAS11* 3′ D3(-) *trans* acting siRNA expression profile triggered by miR482. Right Panel: Expression profile of NB-ARC LRR *VIT*_*12s0034g01460* targeted by *TAS11* 3′ D3(-) tasiRNA. E) *TAS4c* 3′ D4(-) *trans* acting siRNA expression profile (left panel) and the expression profile of *TAS4c* undiced primary transcript (right panel; “NA”: not available. Test samples were normalized to 0 WAV minus UV-B [set to unity]). Error bars are s.d. Asterisks (*) denote significant differences based on analysis of variance (n = 3 biological replicates) and comparisons using the Tukey-Kramer honestly significance test (HSD; *P* < 0.05). Insets, A-E: Target-miRNA/siRNA binding positions (mRNA nucleotide coordinates) and primer binding sites (arrows) are depicted to scale. Target:miRNA binding depicted in Red; Target:tasiRNA binding depicted in Green.

The functional significance of differentially expressed miRNAs upon high UV-B influence in the greenhouse and field was tested by real-time quantitative PCR (qRT-PCR). The expression levels of validated target genes were assayed from the same total RNA samples used to construct the sRNA libraries. The results for greenhouse treatments are shown in Figure 3. An inverse correlation was indicated between the observed miRNA dynamic and some target gene expression profiles during berry development, although the relationship was positively correlated in discrete time points. Differentially expressed miR156f, miR3632-3p (homologous to miR482) and miR530a displayed at certain times of berry development an inverse relationship with their cognate target genes *Squamosa promoter binding protein-like2_3* (Supplementary Table 2a; Supplementary File S4, p. 1), numerous homologs of *VIT_13s0067g00790* encoding Leucine-rich repeat protein (LRR) (Figure 3B; Supplementary Table S2b; Supplementary File S4, p. 66), and *Plus3 Domain* protein-coding genes (Supplementary Table S2a; Supplementary File S4, p. 11), respectively. However, qRT-PCR results for (-) UV-B control samples indicated miR156f likely did not change during development whereas assayed paralogue *SPL2_1* decreased sharply from -3 to 0 (Figure 3A). A similar developmental dynamic was observed for miR530 target *Plus3 Domain* suggesting that UV-B inverse relationships may not be observable before +6 WAV (Figure 3C). A direct relationship was observed between miR482 and derivative tasi-RNA *TAS11* 3′-D3(-) abundance changes during berry development and in response to high UV-B (Figure 3D, left and middle panels). Likewise to miRNA:target dynamics, the *TAS11* regulatory cascade showed evidence of activity against downstream target NB-ARC LRR protein gene (Supplementary Table 2b; Figure 3D, compare middle and right panels). Taken together and in general, the dynamics observed between miRNAs and target expressions suggest that changes in target abundance are mediated transcriptionally and modulated in some cases post-transcriptionally by miRNAs, with the major changes in target abundance controlled primarily by developmental effects that are similar between treatments. miRNAs may act not as dominant negative effectors to drive down target expression (*i.e.*, presenting inverse correlations), but more like a “rheostat” to limit or fine-tune the accumulation of target mRNAs.

Interestingly, a direct concordance was observed in greenhouse high-UV treatments between the tasi-RNA *TAS4c* 3′D4(-) and its primary (undiced) *TAS4c* transcript (Figure 3E), supporting the notion that miR828 induction by high-fluence UV-B throughout berry development (as shown in Figure 2B, D) results in increased *TAS4* expression, marked by the accumulation of *TAS4* phasi-RNAs triggered by miR828 (Figure 3E, left panel; Supplementary Table 2a; File S4, pp. 38-40). Despite their radiation-responsiveness, the UV-induced and developmental trends of *TAS4c*-3′D4(-) and miR828 are opposite to each other (see below). Similar results correlating the apparent decreases in mature miR828 expression after veraison (Figure 2B,D) were observed for pre-*MIR828*, derived miR828* abundance, *TAS4a* 3′D1(+) and *TAS4b* 3′D4(-) tasi-RNAs and their respective primary *TAS4* transcripts from high-fluence UV-B field samples (Supplementary Fig. S2B-E). Despite the very low mature miR828 expressions observed at veraison (Figure 2B, D), its high induction (>threefold change) by UV treatment measured by blot densitometry was also reflected in the normalized abundance of UV-induced miR828* reads and in the UV-induced pre-*MIR828* levels assayed from the same total RNA field samples (Supplementary Fig. S2B). In order to further substantiate these results over developmental time points for a very low-expressed miR828 and derivative effectors, we analyzed the RNAseq datasets of Massonnet *et al.* (2017) for reads mapping to our amplicon pre-*MIR828*, *TAS4*, and *MYBA6/7* targets quantified by qRT-PCR. The results are shown in Table 2 and further corroborate our observations that miR828 (Figure 2B, D) and targets *TAS4ab* (Supplementary Fig. S2) decrease during berry development, as does *MYBA7* (TAS4-3′D4(-)’s target), whereas miR828 target *TAS4c* (Figure 3E, Supplementary Fig. S2E) paradoxically increases during berry development, but remarkably only in red-skinned cultivars (Table 2). This, along with the concordant observation that *MYBA6*-derived tasiRNA expression dynamics (Supplemental Fig. S2F) are associated with higher *MYBA6* expression at post-veraison stages (Table 2), may indicate some unique function of *TAS4c* in *MYBA6* regulation for anthocyanin accumulation.

**Table 2:** Normalized RNAseq quantification of pre-*MIR828*, *TAS4*, and target *MYBA6* and -*MYBA7* mRNAs in berry skins of five field-grown red and white cultivars during development (datasets of Massonnet *et al.* 2017)

Datasets/gene	Berry Developmental Stage			
	Pea-sized	Just prior veraison	Soft	Harvest
**Red cultivars**	(reads per 100 Million*)			
pre-*MIR828*^	1.1	0.0	0.2	0.0
*TAS4a*^	31.4	16.5	6.0	0.4
*TAS4b*^	0.3	0.2	0.0	0.0
*TAS4c*^&^	0.0	0.0	**13.6**	**8.7**
*MYBA6*^$^	5.1	*1.6*	*10.3*	0.9
*MYBA7*^	19.9	13.3	0.0	0.0
pre-*MIR166e* reference^	26.5	20.2	13.2	7.8
**White cultivars**				
pre-*MIR828*^	1.3	0.2	0.0	0.0
*TAS4a*^	53.8	41.7	19.1	3.9
*TAS4b*^	4.6	4.3	0.0	0.0
*TAS4c*	0.0	0.0	0.0	0.0
*MYBA6*^$^	6.0	*3.7*	*5.2*	3.8
*MYBA7*^	8.2	0.0	0.2	0.0
pre-*MIR166e* reference^	23.0	14.4	5.8	3.9

* 96 nt reads mapping completely to amplicons quantified by qRT-PCR in Fig. S2, <= 4 mismatches. See Supplementary Table 7 for amplicon target details. Pre-vvi-*MIR166e* target sequence from miRBase22; *TAS4b* reads are for 105 bp amplicon plus 100 nts upstream and downstream because 96 nt read lengths precluded results.

^ The decreasing pre-*MIR828*, *TAS4ab*, *MYBA7*, and pre-*MIR166e* expressions as development progresses correlate with the derivative miR828*, *TAS4* siRNA, *MYBA7* mRNA, and mature miR166 abundances documented in field samples, Supplementary Table 3d and Supplementary Fig. S2.

^&^The increasing *TAS4c* expression as development progresses **(bold)** correlates with mRNA and derivative *TAS4c* tasiRNA 3′D4(-) abundances documented in field samples, Supplementary Table 3d and Fig. S2E.

$ The fluctuations of *MYBA6* expressions at veraison and soft berry stages (*italics*) correlate with the derivative siRNA abundance documented in field samples, Supplementary Table 3d and Fig. S2F, left panel.

Our findings raised the question of why *TAS4* primary transcript relative abundances are not reduced by high UV-B treatments; *i.e.*, an inverse relationship with miR828 at veraison (Figure 2, Supplementary Fig. S2) as seen for other miRNA targets and tasi-RNA cascades (above). We observed high-fluence UV-B treatments in greenhouse and field significantly delayed the down-regulation of *MYBA6* and *MYBA7* at the latter stages of berry development (Supplementary Fig. S2F, G right panels; Matus *et al.* 2017). We propose, based on these results, that the observed concordant expressions of miR828 (Figure 2, Supplementary Fig. S2B), *TAS4abc* primary transcripts and diced tasi-RNAs (Figure 3E, Supplementary Fig. S2C-E) and the derived *MYBA6* and *MYBA7* phasi-RNAs spawned by the activity of TAS4ab-3′D4(-) (Supplementary Fig. S2F, G left panels; Supplementary File S4, pp. 41, 42), are strong evidence in support of a functional orthologous *MYBA6/7* auto-regulatory loop as described in Arabidopsis (Hsieh *et al.* 2009; Luo *et al.* 2012) acting either directly or indirectly to activate expression of both *MIR828* and *TAS4abc*.

The observed complex regulation of miR828 by UV-B detected by RNA blot and corroborated by sRNA reads and qRT-PCR prompted us to examine other significantly differentially expressed *MIRNA*s (all analyzed by DESeq2) for a pattern of up-regulation by high UV fluence and down-regulation by low UV fluence. Table 3 lists those significant UV-B differentially regulated miRNAs and siRNAs mimicking the pattern (up in high fluence, down in low fluence) observed by RNA blot for miR828 (Figure 2), miR828* and pre-*MIR828* at veraison (Supplementary Fig. S2B). We further endeavored to impute supporting evidence for miRNA function by meta-analysis with respect to the observed UV-B changes of miRNA abundances using co-expression analyses of validated mRNA targets. The subject datasets employed were three genome-wide transcriptomics datasets of grapevine berry skin tissues in response to 1 hr of UV-C exposure (Suzuki *et al.* 2015), five weeks exposure to UV-B after veraison (Carbonell-Bejerano *et al.* 2014), and from five red-skinned varieties sampled across berry developmental stages (Massonnet *et al.* 2017). It is noted that UV-C lamps have about 5% of their energy emitted in the UV-B band (UV-C light source GL-15 lamps have power emission in the UV-B range; http://www.nelt.co.jp/english/products/safl/) supporting our view that these comparisons are worthwhile. Table 3 shows the normalized abundances (ratio of +UV/Control) of selected miRNAs for four time points and three environments in response to UV-B (minus UV-B set to unity), and the reported fold-change effects on validated miRNA target mRNAs (Supplementary File S4; Supplementary Table 2b) after 1 hr UV-C and UV-B treatments. Binomial distribution tests of significance for the observed inverse relationships to occur by chance (a function of number of targets) revealed significant results collectively, and individually for all 10 predicted SPL targets of miR156f, 12 of 14 LRR targets of miR482-triggered *TAS11* 3′D3(-), and in four out of the five validated MYB targets of miR828 (Pantaleo *et al.* 2010) (Supplementary Table 2b; Supplementary File S4, pp. 44-48). Taken together these results are consistent with the hypothesis that high-fluence UV-B can induce miRNA accumulations that consequently have an effect in decreasing berry skin target gene expressions.

**Table 3:** Co-expression analysis of high-fluence UV-B induction of miRNAs/phasi-RNAs abundances during berry development which are inversely correlated with predicted target mRNA expressions in the same skin tissues in response to UV-C pre-veraison and UV-B five weeks post-veraison from independent experiments (Suzuki *et al.* 2015; Carbonell-Bejerano *et al.* 2014)

		UV-B Fold Change^&^ (bold= up; *italics* = down)												Inverse correlation of mRNA target expression *vs.* miRNA/siRNA?			
		High-Fluence Greenhouse				Low-Fluence Greenhouse				High-Fluence Field							
miRNA/siRNA	LFC, UV-B	-3 WAV	Verai-son	+3 WAV	**+**6 WAV	-3 WAV	Verai-son	**+**3 WAV	**+**6 WAV	-3 WAV	Verai-son	**+**3 WAV	+6 WAV	Validated Targets* (n; annotation)	FC, UV-C^$^	FC, UV-B**†**	FC *P*-val^^^
miR156f	0.68	**1.332**	**2.755**	**3.266**	**5.520**	*0.644*	10.25	1.863	*0.567*	**1.440**	**2.149**	**1.144**	**1.430**	11; SPB	**0.75**	0.89	0.02
miR530b	−0.77^+^	n.d.	**2.556**	0.488	**1.183**	*0.258*	*0.853*	*0.200*	*0.065*	n.d.	**5.908**	**5.853**	**6.316**	2; Plus3, DYW	**0.80**	0.86	0.09
miR477c	−0.32^+^	0.010	**3.253**	**3.129**	**2.772**	*0.017*	*0.104*	*0.116*	*0.003*	**3.082**	**1.177**	**3.039**	0.374	2; GRAS-domain	n.d.	0.84	0.51
*TAS4a*, miR828 target	0.32	0.561	**3.540**	**5.119**	**1.249**	*0.102*	2.155	*0.554*	*0.357*	**2.772**	**2.602**	**1.961**	**1.192**	2; MYBA6/A7	**0.63**	0.97	0.30
*TAS4b*, miR828 target	1.10	0.089	**1.704**	**2.115**	**60.16**	1.006	2.713	2.562	*0.572*	**4.956**	**7.022**	**6.213**	**2.953**				
*TAS4c*, miR828 target	0.14	**5.381**	**4.825**	**1.712**	0.955	*0.180*	1.348	1.342	*0.513*	0.318	**1.777**	**1.953**	0.750				
miR828 target MYB, phasi-RNA producing	−0.29^+^	0.218	**1.425**	**1.023**	**1.725**	*0.130*	2.948	1.260	*0.377*	**1.151**	**1.367**	0.569	**1.316**	5; MYBs	**0.70**	0.87	0.04
miR482	0.07	0.340	0.965	**1.188**	**2.936**	*0.393*	2.583	2.840	*0.624*	**1.450**	**1.678**	0.774	0.855	2;*TAS11*,slyTAS5-Like	**0.80**	0.96	0.09
*TAS11*-D3(-) miR482 target	0.17	0.287	**1.467**	**1.290**	**5.191**	*0.515*	5.015	1.697	*0.419*	**1.673**	**1.167**	0.733	**1.279**	14; LRRs	**0.84**	0.86	0.02
miR403a-e average	−0.19	0.504	**1.206**	0.205	**1.212**	2.764	2.238	*0.347*	*0.382*	**1.566**	0.987	0.678	**2.044**	1; AGO2	1.1	1.07	0.17
														Overall 38 targets, two experiments			5.3E-6

^&^ Normalized to 20M reads (+1 if zero reads). Denominator is corresponding normalized -UV-B sample (unity). n.d.: not detected.

+ sign negative overall because low-fluence UV-B effect dominant, or low read counts independently filtered out by PhaseTank.

* See Supplementary Table 2a.

$ Data for 1 hr FC by UV-C treatment of pre-veraison berries from Suzuki *et al.* (2015). If called significantly different expression in response to UV-C, in **bold**.

^ Binomial distribution probability for FCs of al target mRNA responses to UV-C and UV-B inverse to miRNA/siRNA (up) under high-fluence UV-B.

† Data for FC by UV-B treatment on 26 °Brix (ripe) berries irradiated for five weeks post-veraison from Carbonell-Bejerano *et al.* (2014). For miR403 target AGO2, includes 23°Brix sample (3 independent tests for significance).

### UV-B regulated miRNAs with different fluence-rate responses

Based on the apparent coordination for miRNA expressions by high- *vs.* low-fluence UV-B treatments, the high- and low-fluence irradiated greenhouse berry samples were tested against each other (high/low ratio across developmental time, including across (-) UV controls). The field UV-B irradiated berries were not included in the test since the environmental variables were likely confounding *vs.* within-greenhouse comparisons. Supplementary Table 3c lists 18 miRNAs with significant differential expression by high-fluence UV-B treatment *per se*, of which 11 were up-regulated and seven were down-regulated. Three novel miRNAs (called by ShortStack as valid *MIRNA*s or missing only evidence for the star species) were identified to be differentially expressed in response to high-fluence UV-B. We found multiple candidate family members of vvi-*MIR477* of which miRBase has only two annotated: miR477a and miR477b. The five others that respond differentially to UV-B high *vs.* low fluence are listed in Supplementary Table 3c and are presumed to be encompassed by the nine vvi-miRC477c,i-p candidate family members previously described by Paim Pinto *et al.* (2016). Similar to the UV-B response results across greenhouse and field berry samples (Supplementary Table 3b), vvi-*MIR3627* was highly up-regulated under high-fluence UV-B (Supplementary Table 3c). vvi-*MIR3627* is an atypical *MIRNA* locus in that it produces phased sRNAs and is not stranded (it produces abundant antisense transcripts; Supplementary Table 3b). vvi-MIR399i, which is predicted to target phosphate transporters (Supplementary Table 2a) (Mica *et al.* 2010), and vvi-MIR167c, targeting *ARF* TFs (Supplementary Table 2a; Supplementary File S4, p. 9), were some of the miRNAs up-regulated by high-fluence UV-B *vs.* low-fluence reference samples.

Two interesting observations were the coordinated downregulation of all six miR403 family members (five of them significantly) by high-fluence UV-B, that target *ARGONAUTE 2* (*AGO2)* in dicots (Supplementary Tables 2a, 2b; Supplementary File S4, p. 34). *AGO2* is correspondingly up-regulated by UV-C and UV-B (Table 3) and associated with stress responses including DNA damage (Fátyol *et al.* 2016; Wei *et al.* 2012). The up-regulation of *MIR403f* primary transcript during berry maturation in field samples (Supplementary Fig. S3) validated our observation of significant up-regulation across developmental time points for miR403 species (Supplementary Table 3d). *TAS4a* targeted by vvi-miR828 (phase Score: 18,631; Supplementary Table 2a; File S4, p. 38), a miR828-targeted MYB (phase Score: 1,260, Supplementary File S4, p. 47), and a miR482/Vv-sly-TAS5-like-3′D6 (+)-triggered LRR PHASI locus (phase score 398.3; Supplementary Table 2b) were significantly up-regulated by high-fluence UV-B, consistent with observed trigger miRNA abundances (Figure 2, Supplementary Table 3c). However, *TAS4*c (which has a 3′D4(-) variant in our cv. ‘Cabernet Sauvignon’ sRNA libraries that targets *VviMYBA6/7* less well due to a mismatch at seed position 10; Supplementary Table 2b), despite evidence for being up-regulated by high-fluence UV-B *per se* (like *TAS4ab*; Table 3), was expressed highly in both control and low-fluence UV treatments compared to the high-fluence conditions (not shown), resulting in an overall negative coefficient for high-fluence effect like seen for miR403 family members (Table 3; Supplementary Table 3b,c). These results suggest an additional regulatory mechanism affecting *TAS4c* expression, which we speculate might involve the TAS4c-3′-D4(-) mismatches to target *MYBA6/A7* impacting a functional interaction with the hypothesized auto-regulatory loop.

### Small RNAs differentially expressed during berry development

To characterize the overall accumulation of miRNAs and siRNAs affected by berry ripening, sRNA libraries constructed from berries (-3, 0, +3 and +6 WAV) harvested from greenhouse and fields were tested for differential expression across developmental time using the likelihood ratio test. By assuming (null model) there is no difference in miRNA expression profiles between different berry development stages, we identified 48 miRNAs to be significantly differentially expressed at one or more developmental time points across environmental treatments (Supplementary Table 3d). As expected for phenotypic changes associated with veraison, we observed profound dynamics in miRNA abundances. 21 miRNAs were up-regulated while 27 displayed a significant negative log fold-change during development. Linear regression (Pearson coefficient; Supplementary Table 3d) as a metric to correlate time (three equally spaced time points before berry maturity at harvest) with normalized miRNA/siRNA abundances demonstrated a strong linear correlation across the developmental time points with replicates (Supplementary Table 3d). This strong linear relationship held for nearly every *MIRNA* and PHASI locus even when fold changes were small and non-significant. This evidence demonstrates that the experimental parameters were highly reproducible and the results warrant inference of miRNA/siRNA functions toward validated targets as shown above. We performed co-expression analysis of our differentially expressed (DE) miRNAs (Supplementary Table 3d) with fruit developmental RNA-Seq datasets from Massonnet *et al.* (2017) that reported significantly DE mRNAs at four comparable time points in five red-skinned varieties. We identified 15 cases of miRNA/target mRNAs with statistically significant (binomial test of chance) inverse correlations, supporting functional effects of miRNA changes during development (Supplementary Tables 2b, 3d).

miR530ab may target 3′ UTR intronic regions of *AGO1* orthologs, supported by GSTAr alignment data (Supplementary Table 2a). miR393ab, which degradome evidence shows triggers phasi-RNA production and phasi-RNA auto- and *trans* cleavages (Si-Ammour *et al.* 2011) in *AUXIN TRANSPORT INHIBITOR RESPONSE1/TIR1* co-receptor mRNAs (Supplementary Tables 2a, 4; Supplementary Files S5, S4 p. 20), were also coordinately up-regulated during berry development (Supplementary Table 3d).

As shown previously, *TAS4a*, *-b* and *-c* siRNAs (triggered by miR828), that in turn trigger phasing of *MYBA6* and *MYBA7* siRNA production via *TAS4ab*-3′-D4(-), showed differential expressions during berry development. *TAS4a* siRNAs expression was maximal at -3 WAV, and decreased at veraison, +3 WAV and +6 WAV (Supplementary Table 3d). *TAS4b* siRNAs displayed a lower expression than *TAS4a*, and also decreased along developmental stages. Interestingly, *TAS4c* reads increased at every developmental stage when compared to the previous stage (Supplementary Table 3d), consistent with the observation that *TAS4c* and miR403 were regulated differently than *TAS4ab* and most miRNAs (Supplementary Table 3c), which decrease during berry development (Supplementary Table 3d).

## Discussion

We have endeavored to characterize the miRNA and phasi-RNA space in response to UV-B light by target degradome validations, statistical inference of sRNA dynamics, and direct qRT-PCR measurement and meta-analysis of miRNA and target mRNA abundances in published datasets from the same organs and tissues. Shortstack ver3, by virtue of its multimapper functionality (Johnson *et al.* 2016), was our method of choice for quantifying locus-specific miRNA precursor (*i.e.*, cluster) abundance as a proxy for mature miRNA and phasi-RNA levels. This approach presumes that when a sRNA cluster is called as differentially expressed, the mature miRNA (or most abundant phasi-RNA) is the species changing in response to a treatment or condition. Even if the subject sRNA ostensibly undergoes dynamic fluctuations in abundance, it cannot function as a negative effector of target gene activity without being loaded in a functional AGO-containing RISC, which along with base pairing to target mRNAs, finally controls miRNA/tasiRNA stability, activity, and subcellular retention (Li *et al.* 2013; Pitchiaya *et al.* 2017; Speth *et al.* 2013). It is remarkable that miR828, a 22 nt species with a very low estimated abundance (the sequenced star species = 2.6 rpm from 411 raw reads, meaning miR828 was sequenced only a few times across all libraries ∼0.01 rpm), manifested the highest *in vivo* slicing activities based on phasi-RNA abundances (∼15,000 rpm) and phasing scores from *TAS4abc* and most of the miR828 target *MYB*s. Single nucleotide polymorphisms of sRNAs across mapping datasets could also be a confounding factor in interpreting our differentially expressed candidate miRNAs. Caution is warranted when interpreting phasi-RNA abundances (*e.g.*, *TAS4a,b,c*) where there are many species generated, some of which can accumulate to high levels yet only one (*e.g.*, *TAS4* 3′-D4[-]; Rock 2013) is claimed as functional. The functional siRNA and miRNA may not be the major species in clusters or the one that changes significantly in response to stimulus, confounding interpretations. For *TAS4a*, the major species called by ShortStack is documented as 3′D1(+) (Supplementary Table 3d, column “I”). However, Supplementary Table 3d and Supplementary File S4 (p. 38) provides direct evidence supporting the assumption that the claimed differentially expressed mature miRNAs and phasi-RNAs (*e.g.*, *TAS4b,c* 3′D4[-]) are the major species changing their abundances *per se*.

Initial prediction of UV-B-responsive miRNAs in Arabidopsis was founded on computational analyses of UV-regulated co-expressed genes, which shared promoter motifs with candidate *MIRNA*s, coupled with predicted target expression that inversely correlated with inferred miRNA profiles (Zhou *et al.* 2007). UV-B responsive miRNAs were later identified in poplar (Jia *et al.* 2009), wheat (Wang *et al.* 2013), maize (Casati 2013), and *Brassica rapa* in relation to UV-A (Zhou *et al.* 2016). In maize, miR156 and miR529 are decreased while their several targets *Squamosa Promoter-Binding-like* (*SPL* transcripts) are increased after 8 hr of UV-B exposure of maize leaves (Casati 2013). Wang *et al.* (2013) also reported down-regulation of miR156 by UV-B in wheat. We also observed a few inverse correlations between vvi-miR156 abundances and *SPL* target expressions in our experiments which were consistent with published datasets for UV-C and UV-B responses (Table 3, Supplementary Table 2b), but in contrast to maize and wheat we found vvi-miR156f and vvi-miR535c to be up-regulated in grapevine in response to UV-B. It is possible that the low-fluence down-regulation effect on numerous miRNAs (including miR156f; Table 3) could account for the observed differences between grape and maize or wheat, where a coordinated antagonist response pathway for low-fluence UV-B may be involved. Previous work on grapevine leaves showed high-fluence rate UV-B specifically modulates pathways and processes associated with oxidative and biotic stress, cell cycle progression, and protein degradation, whereas low-fluence rate UV-B promotes photomorphogenic responses and regulates the manifestation of auxin, ABA, and cell wall modification processes (Pontin *et al.* 2010). Consistent with this notion is the differential UV-B fluence effects on *VviTAS3* phasi-RNAs and miR828 abundances (up in high-, down in low-fluence), and with prior work showing *TAS4* is negatively regulated by ABA/sugar crosstalk signaling (Luo *et al.* 2012). The higher expression level and lack of UV-B induction of miR828 or pre-*MIR828* in *in vitro*-grown plantlets (Figure 2A; data not shown) in contra-distinction to berries is consistent with a possible negative effect of increased ABA in leaves (Loveys 1984).

### A bimodal pathway operates under low-fluence UV-B regulating photomorphogenesis

**Figure 4:**
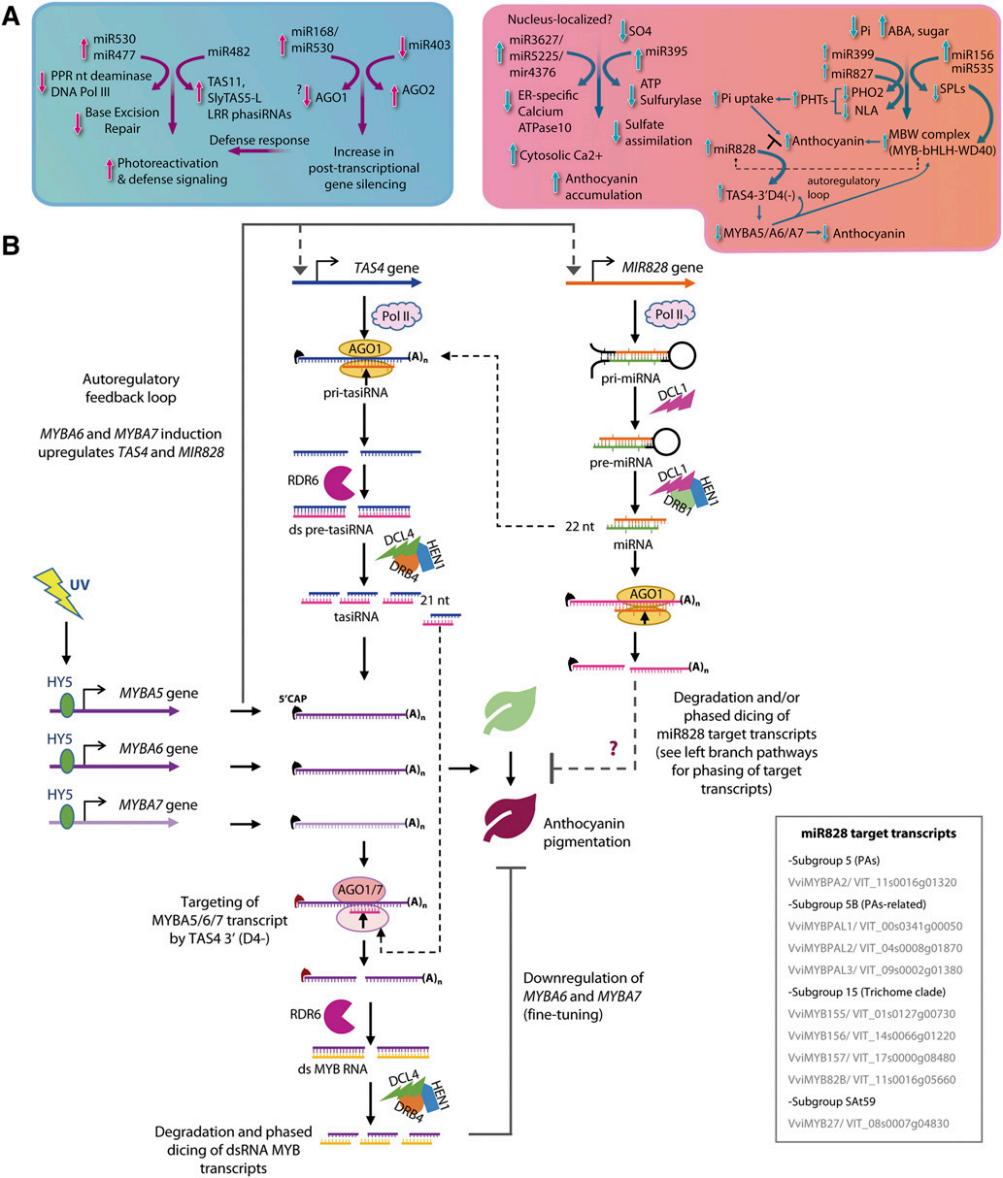
A) Model of UV-B effects on grape berry stress responses and development regulated by miRNAs. Defense, DNA repair and accumulation of anthocyanin are the major responses regulated by UV-B responsive miRNAs. Increase in miR168/miR530 facilitate post-transcriptional gene silencing activity. The increase in PTGS activity would in turn facilitate targeting of *LRR* genes by miR482 and regulate the defense genes. miR477 family members target *DNA Pol III* involved in base excision repair (BER) and facilitates photo-reactivation and nucleotide excision repair (NER) to remove UV-B induced DNA lesions. Upregulation of miR3627/4376/5225 by UV-B targets ER-specific *Calcium ATPase10* and facilitate increase in cytosolic Ca^2+^ levels and thereby increased anthocyanin accumulation. miRNAs shown to be regulated by sulfur (miR395) and phosphorous starvation (miR399; miR827) are also responsive to UV-B radiation and facilitate anthocyanin accumulation. miR156/miR535 target *SPL* genes and thereby increase the accumulation of MYB-bHLH-WD40 TFs and anthocyanin as elucidated in Arabidopsis. Increase in MYB-bHLH-WD40 TFs would trigger the conserved auto-regulatory loop involving miR828/*TAS4* to regulate *MYBA6/A7* levels and thereby anthocyanin levels. B) Schematic representation of the auto-regulatory feedback loop of miR828/TAS4 exerted by MYBA genes from chromosome 14.

UV-B signaling mechanisms affecting grape berry polyphenolics (flavor enhancers and fruit astringency components) have been previously characterized (Loyola *et al.* 2016; Martínez-Lüscher *et al.* 2013), but there is no description of grapevine UV-B responsive miRNAs and their significance for regulation of polyphenolic biosynthesis, stress responses, or berry development. Our analysis reveals a dynamic regulation of certain miRNAs following UV-B-induction and pervasive changes in miRNA profiles during fruit development. We have formulated a model (Figure 4) to frame high-fluence miRNA dynamic responses to UV-B in the context of post-transcriptional gene silencing processes impacting oxidative and nutrient (sulfur and phosphate, associated with anthocyanin accumulation) stress adaptation during grape berry development. We suggest that these high-fluence UV-B response processes may be different between dicots *vs.* monocots, like the case for *MIR828/TAS4* regulation of anthocyanin biosynthesis (Rock 2013) based on three grounds: i) observed differences between our results for deeply conserved and functionally significant miR156 changes (Figure 3, Table 3) in contradistinction of results in maize and wheat (Casati 2013; Wang *et al.* 2013). ii) The proposed coordinate antagonist response pathway for UV-regulated miRNAs revealed by the low-fluence UV-B experiment (Figure 2, Table 3) affecting predominantly dicot-specific miRNAs (Supplementary Table 3a-c). iii) Differences in overrepresented gene ontology categories between eudicots and monocots suggest the evolution of oxygen and radical detoxification (*i.e.*, UV associated) and RNA silencing processes mark key divergence points between dicots and monocots (Lee *et al.* 2011). Our results suggest no overlap between the miRNAs regulating photomorphogenic and defense pathways in response to low-fluence UV-B, in accordance with those of Yoon *et al.* (2016) and Pontin *et al.* (2010), who showed low levels of UV-B irradiation result in photomorphogenic and auxin responses rather than induction of defense- and abiotic oxidative stress-related genes.

### Polyphenolic pathway and cellular transcription are targets of UV-B responsive miRNAs

Frohnmeyer *et al.* (1999) demonstrated that UV-B triggers an increase in free cytosolic Ca^2+^ levels associated with UV-B induction of *CHS*, and Loyola *et al.* (2016) demonstrated UV-B induction of *CHS3* in irradiated grapevine plantlets. Calcium transporting ATPase10 is known to be involved in transporting free cytosolic Ca^2+^ to the endoplasmic reticulum (Geisler *et al.* 2000). The evolutionarily related miR5225, miR3627, and miR4376 (offset by 4 nt) were up-regulated upon UV-B irradiation. As they all target the 5′ UTR intron2 region of *Calcium-transporting ATPase10* pre-mRNA (Supplementary Table 2a; Supplementary File S4, pp. 56-57), we propose these miRNA family members as modulators of free cytosolic Ca^2+^ levels and *CHS* expression (Figure 4A).

Considering that nutrient availability impacts on miRNA networks regulating anthocyanin metabolism, vvi-MIR399i, which targets *PHO2* (a ubiquitin-conjugating E2 enzyme regulating the stability of phosphate transporters) is up-regulated by high-fluence UV-B. *PHO2* repression by miR399 in response to UV would affect negatively P_i_ uptake (Lin *et al.* 2008) (Figure 4A). Consistent with this model, phosphate deficiency increases anthocyanin content in grapevine cell cultures (Yamakawa *et al.* 1983).

vvi-MIR395 is a 5′ RLM-RACE-validated slicer of *ATP Sulfurylase 1*supported by GSTAr alignment prediction, which showed a strong and significant inverse correlation between miRNA and target mRNA abundances in response to cold stress in grapevine (Sun *et al.* 2015). ATP Sulfurylase, along with validated miR395 target *AST68*, a low affinity sulfate transporter (Supplementary Table 2a, File S4 p. 22), functions to increase sulfur transfer to aerial parts under sulfur starvation (Kawashima *et al.* 2011). MIR395 up-regulation in response to drought stress in rice (Zhou *et al.* 2010) and salinity stress in *Zea mays* (Ding *et al.* 2009) is consistent with our evidence for a role in abiotic stress responses including UV-B. Sulfur starvation increases anthocyanin levels in rice (Lunde *et al.* 2008) and in grapevine sulfur- dependent oxidative response effectors GLUTATHIONE S TRANSFERASE (GST) and glutaredoxin are induced during veraison (Terrier *et al.* 2005). Different GSTs have been recently related to flavonoid transport (Pérez-Díaz *et al.* 2016), with VviGST1/4 having major roles in anthocyanin transport (Conn *et al.* 2008; Gomez *et al.* 2011).

UV-B induced the accumulation of vvi-MIR535c and vvi-MIR156f, the latter of which we show and impute (Table 3) to coordinately down regulate *SPL* transcripts. Up-regulation of miR535 also may contribute to observed down-regulation of *SPL*s, although a bulge at nt 6 of the seed region and lower degradome p-values qualify this assertion (Supplementary Table 2a; 2b). Several groups have reported *SPL* transcripts in grapevine targeted by both miR156 and miR535 (Carra *et al.* 2009; Han *et al.* 2016; Pantaleo *et al.* 2010). *SPL* transcript reduction increases anthocyanin levels in Arabidopsis (Gou *et al.* 2011) and our functional co-expression analysis supports that UV-B regulation of miR156 is an important component of a regulatory network for anti-oxidant and UV-protective polyphenolic biosynthesis (Figure 4A). The preponderance of transcriptional regulators and TAS/PHASI targets of UV-B regulated miRNAs in grapevine and across species supports a model integrating nodes of conserved regulatory networks important for integrating abiotic stress and developmental programs.

### Phenylpropanoid pathway and defense genes are targets of UV-B responsive tasiRNAs

Recent papers report a role of miR858 in Arabidopsis related to immunity upon nematode and fungal pathogen infections by negative regulation of flavonoid-specific *MYB* regulators (Camargo-Ramírez *et al.* 2018; Piya *et al.* 2017). Other researchers have invoked the ’two-hit’ model to explain observed phasi-RNA production of miR828- and miR858-dual targeted *MYB*s involved in proanthocyanidin and anthocyanin biosynthesis, based on weak predicted binding affinity of miR858 in a conserved domain 12 nt upstream of miR828 binding sites (Guan *et al.* 2014; Xia *et al.* 2012; Zhu *et al.* 2012). Several grapevine *MYB*s with a conserved miR858-miR828 dual binding domain as described in peach, apple, cotton, and Arabidopsis (Guan *et al.* 2014; Xia *et al.* 2012; Zhu *et al.* 2012) showed compelling evidence of slicing by miR828 that spawns phasi-RNAs in register. However, these targets only showed marginal evidence of miR858 slicing that correlates with high (4.5- 6.5) Allen scores (Supplementary File S4, pp. 44, 47, 49, 50). Although it is plausible that dual auto-regulatory loops for miR828:*MYBA6/7* (Rock 2013) and miR858:*MYB82* (Piya *et al.* 2017) operate coordinately in grapevine to regulate anthocyanin and flavonol biosynthesis, respectively like in Arabidopsis, our functional analysis supports miR828 being the dominant effector module. It is noteworthy that for *MYBA6/7* slicing by 21 nt TAS4ab-3′D4(-) and production of abundant phased registers downstream, there is no obvious trigger mechanism like described for the ’two-hit’ and 22_1_ hit miRNA models for phasi-RNA production (Axtell *et al.* 2006).

Primary *TAS4* transcripts, miR828 and *TAS4abc* 3′D4(-) tasiRNAs triggered by miR828 are up-regulated by high-fluence UV-B in greenhouse and field, which is in line with the function of a conserved auto-regulatory feedback loop proposed by Hsieh *et al.* (2009) and Luo *et al.* (2012). Increases in VvMYBA6/7 induce *TAS4* (and *MIR828*) transcription and thereby trigger a feedback repression of *MYB* genes targeted by miR828 and *TAS4* (Figure 4B).

Novel *TAS11* and vvi-sly*TAS5*-like loci are triggered by miR482 up-regulation under high-fluence UV, generating several phasi-RNAs from multiple registers. These in turn target numerous *LRR* genes, which themselves undergo UV-B dependent siRNA amplification (Supplementary Table 3a-c) and subsequent functional silencing (Table 3, Figure 3D; Supplementary Table 2b; Supplementary File S4, p. 37). The repression of defense genes by miRNAs until the plant is exposed to severe stress is a hypothesized adaptive mechanism to keep the defense response under post-transcriptional control to conserve energy by preventing LRR protein synthesis (Shivaprasad *et al.* 2012). A domain of the nuclear Armadillo-type found in Cap Binding Protein Large Subunit/CBP80 and LRR variants is targeted by D6(+) phase of vvi-sly*TAS5*-like, based on degradome evidence. Mutations in *cap binding protein20* and *cbp80* are ABA hypersensitive, drought tolerant, and have abnormal miRNA biogenesis (Gregory *et al.* 2008; Papp *et al.* 2004). It is plausible that targeting of a cap binding complex by elevated miR482/*TAS* phasi-RNA expression may be an adaptive mechanism for UV-B response/tolerance and a crosstalk mechanism linking UV-B fluence dynamics of miRNAs (Table 3) to ABA antagonism of *MIR828/TAS4* feedback homeostasis (Luo *et al.* 2012).

### Oxidative stress by high-fluence UV-B combated by miRNAs?

The 18 differentially expressed miRNAs identified as changing under high *vs.* low-fluence *per se* were more than found when UV-B radiation across all environments (including field trials) was tested as a single parameter (Supplementary Table 3b). This is possibly because of the noise associated with different (*i.e.*, field) environments. However, Pontin *et al.* (2010) observed the same degree of increased effects of high- *vs.* low-fluence UV-B changes in a grapevine leaf transcriptome comparison. One upshot from the alternative perspectives afforded by the controlled greenhouse experiments was the finding that certain miRNAs/siRNAs (*e.g.*, miR403, miR530, *TAS4c*) are regulated differently and possibly coordinately by UV and development. Six miRNAs (five of them novel; Supplementary File S6) belonging to the *MIR477* family were up-regulated by UV-B and had star species that were much more abundant. vvi-miR477b is predicted to uniquely target *RepC/DNA Pol III epsilon subunit τ* (Supplementary Table 2b) associated with nucleotide excision/mismatch repair pathways. Interestingly, *MIR477*, *MIR482*, and *MIR3627* are conserved across woody fruit trees (Solofoharivelo *et al.* 2014) including grapevine. We speculate that the prevalence of vvi-MIR477 family members induced by high-fluence UV-B may indicate a regulatory role in countering oxidative stress. UV-B radiation is known to induce cyclobutane pyrimidine dimers that kill cells by blocking DNA replication (Ries *et al.* 2000). Photorepair by photolyase and nucleotide excision repair (NER) are the major pathways in plants for repairing UV-B-induced DNA damage (Tuteja *et al.* 2001), while base excision repair (BER) involving RepC/DNAPol III epsilon is involved in repairing oxidized or hydrated bases and single-stranded breaks caused by ionizing radiation (Gill *et al.* 2015). We speculate that the observed up-regulation of vvi-*MIR477* family members may represent an adaptation to reduce BER mechanisms and facilitate photoreactivation and NER, thus removing UV-B induced DNA lesions (Figure 4A).

Liang *et al.* (2013) demonstrated that in addition to miR168, AGO1 is a target of miR530 in papaya and a predicted target of miR530 in *Salvia miltiorrhiza* (Shao and Lu 2013). We predict that two grape *AGO1* orthologs could also be targets of vvi-miR530-isomiR. Harvey *et al.* (2011) demonstrated AGO2 acting as a second layer of control in antiviral defense that is activated when AGO1 is suppressed. When AGO1 is active it represses *AGO2* via miR403 slicing. Interestingly, AGO2 induction by *Pseudomonas syringae* pv. *tomato* bacterial infection without suppression by its negative effector miR403 was observed in the *ago1-27* mutant, suggesting a post-transcriptional regulation of *AGO2* by bacteria, which is mechanistically different from the induction triggered by viruses (Zhang *et al.* 2011). Our assay of *AGO2* transcript abundances in samples from low- and high-fluence UV-B greenhouse-treated berries showed inverse trends to documented miR403 dynamics, but results were not statistically significant. Based on the observed coordinate down-regulation of all vvi-miR403 family members and imputed up-regulation (albeit not statistically significant; *P* = 0.17) of validated target *AGO2* in response to UV-B (Table 3; Supplementary Table 3c; Supplementary File S4, p. 34), we speculate on a third component to the model that might operate in UV-B-radiated grapes in which AGO2 could play a role in combating UV oxidative stress without inactivation of AGO1 (Figure 4).

### Auto-regulatory loop involving miR828-TAS4-MYBA6/A7/A5-MYB113-Like is conserved in berry development

Our specific rationale for studying miRNAs/phasi-RNAs is their involvement in phenylpropanoid accumulation in relation to pigmentation, one of the most important traits for grape berry ripening and subject to nutrient and phytohormone effects (Vanhaelewyn *et al.* 2016). UV-B radiation reduces berry weight and increases anthocyanin content (Berli *et al.* 2010; Berli *et al.* 2011). In addition to phosphate deficiency (Yamakawa *et al.* 1983), rises in ABA concentration and biosynthesis/response effectors such as MYBPA, ATHB-12, ABI5, Nine-*cis*-epoxycarotenoid dioxygenase4, and PAP1/PAP2 increase anthocyanin levels (Hiratsuka *et al.* 2001; Koyama *et al.* 2010; Luo *et al.* 2012). Interestingly, UV-B radiation does not affect berry ABA content whereas leaves exposed to UV-B increase ABA levels (Berli *et al.* 2010; Berli and Ruben 2013). The vegetative anthocyanin loci *MYBA6*, *MYBA7*, and presumably *MYBA5/MYB113-like* and their regulator *HY5* are up-regulated during early berry development when exposed to UV-B and despite expression declines with berry ripening, this behavior is delayed in response to UV (Supplementary Fig. S2F,G; Loyola *et al.* 2016; Matus *et al.* 2017). Pilati *et al.* (2017) reported up-regulation of *MYBA7* when berries are treated with ABA for 20 h but not when the treatment was prolonged to 44 h. This observation is consistent with the notion that *MYBA7* is induced directly by ABA but does not have a role in berry ripening *per se*. vvi-*MIR828* and *TAS4* are validated in this work to regulators of expression of *MYBA6*, *MYBA7*, and *MYB5/MYB113-like*. The auto-regulatory feedback loop is shown to function in grapevine in a similar fashion to Arabidopsis (Figure 4B; Hsieh *et al.* 2009; Luo *et al.* 2012), whereby developmental induction (early in berry development) of MYBA6 and MYBA7 correlates with observed elevated miR828 and high-fluence UV-B inductions at the green berry and veraison stages. In addition, *MYBA* gene expression is concordant with temporal accumulations of *TAS4abc* primary transcripts and derivative siRNAs.

Interestingly *TAS4c* 3′D4(-), which in our analysis of cv. ‘Cabernet Sauvignon’ had two different single nucleotide polymorphisms compared to *TAS4ab* and the reference genome sequence for *TAS4c*, displayed a trend of increasing reads abundance with advancement of berry ripening. This result is intriguing because earlier studies on *Vitis amurensis* and *Vitis vinifera* (cv. ‘Pinot Noir’) grape datasets could not detect appreciable *TAS4c* phasi-RNA expression (Rock 2013), and here we show (Table 2) that green-skinned cultivars do not express *TAS4c*. It will be critical to ascertain if the trait of *TAS4c*-3′D4(-) functional variant expression late in berry development is unique to cv. ’Cabernet Sauvignon’, or if it constitutes a key regulator of a branch pathway in red cultivars for polyphenolic biosynthesis, or a specificity determinant for the MYBA6/A7/A5-MYB113-like autoregulatory loop. *MYBA6* and *MYBA7* phasi-RNAs triggered by TAS4ab-3′D4(-) corresponding to the sixth phase are the most abundant (Rock 2013). Similar to earlier work we find here that the sixth phase of *MYBA6/7* phasi-RNAs are the major species where *MYBA7* phasi-RNA D6(+) is significantly down-regulated during berry development, in stark contrast to *TAS4c*3′-D4(-) significant up-regulation. Further evidence consistent with the functioning of this feedback loop comes from RNA-Seq transcriptome data for several red-skinned berries (Table 2; Massonnet *et al.* 2017). The significant up-regulation of *MYBA6* mRNA in the pre-veraison-to-veraison transition is *inversely* correlated with the observed down regulation of *TAS4ab* and downstream *MYBA6/7* phasi-RNAs, suggesting a positive feed-forward activity for the TAS4ab 3′D4(-) tasi-RNAs slicing *MYBA6/7* and *MYBA5/MYB113-like*. The subsequent wholesale inversion of the *MYBA6* target mRNA:*TAS4a* and *TAS4b* primary transcripts and tasi-RNA relationships (*i.e.*, both significantly down) at berry maturity is parsimonious with the predicted silencing functions of TAS4ab-3′D4(-) on *MYBA6/7* late in berry development. Matus *et al.* (2017) showed high level expression and significant UV-B induction of *MYBA6/7* before veraison and Table 2 shows complete down-regulation of *MYBA7* (but not *MYBA6*, which was delayed per Matus *et al.* 2017*)* during and after veraison.

Our results of differential UV-B fluence effects on miR828 (Figure 2B-D) and pre-*MIR828* (Supplementary Fig. S2B) and developmental stage-specific *TAS4a-c* expressions (Supplementary Table 3b, d) suggest that the auto-regulatory circuit may function to fine-tune expression of *MYBA6/7* in response to UV-B and during berry skin maturation. Our results reveal complex activities of *MIR828/TAS4* in response to environmental and developmental queues to post-transcriptionally silence effectors and underscore the importance of post-transcriptional controls in the observed delays by UV-B exposure on repression of *MYBA6* late in berry development. Understanding the significance of miRNA/phasi-RNA silencing of target mRNAs awaits additional data such as rates of PolII transcription, splicing, polyadenylation, RNA-templated antisense transcription, dicing, slicing, and exosome degradation of target mRNAs. Our exploratory results and meta-analysis provide a basis for systems approaches to better understand non-coding RNA functions in response to UV light and strategies for improving wine grape production of anti-oxidant flavor enhancers.
